# Advanced Material Strategy for Restoring Damaged Endodontically Treated Teeth: A Comprehensive Review

**DOI:** 10.3390/ma17153736

**Published:** 2024-07-28

**Authors:** Elisa Caussin, Mathieu Izart, Romain Ceinos, Jean-Pierre Attal, Fleur Beres, Philippe François

**Affiliations:** 1 Faculty of Dental Surgery, University of Paris Cité, 75006 Paris, France; 2 Bretonneau Hospital, Assistance Publique des Hôpitaux de Paris, 75018 Paris, France; 3 URB2i, Université of Paris Cité, 92100 Montrouge, France; 4 Faculty of Dental Surgery, Côte d’Azur University, 06000 Nice, France; 5 Charles Foix Hospital, Assistance Publique des Hôpitaux de Paris, 94200 Ivry-Sur-Seine, France

**Keywords:** endodontically treated teeth, prosthodontics, indirect restoration, resin composite, post and core

## Abstract

The restoration of endodontically treated teeth (ETT) remains a significant challenge in modern dentistry. These teeth often suffer from substantial structural damage due to both the original pathology and the invasive nature of endodontic procedures. Consequently, ETT are more susceptible to fractures compared to vital teeth, necessitating restorative strategies that can effectively restore both function and aesthetics while minimizing the risk of failure. In recent years, advances in adhesive dentistry and the development of high-strength ceramics have further expanded the restorative options for ETT. Bonded restorations have gained popularity as they preserve more tooth structure and enhance the overall strenght of the tooth-restoration complex. The choice of restorative material and technique is influenced by numerous factors, including the amount of remaining tooth structure, the functional requirements of the tooth, and the aesthetic demands of the patient. Despite the plethora of available materials and techniques, the optimal approach to restoring ETT remains a topic of ongoing research and debate. In this comprehensive review, the current state of and recent advances in restoring damaged endodontically treated teeth are explored. Numerous therapeutic options exist, involving a wide range of materials. This article aims to present the biomaterial advancements of the past decade and their applications, offering alternative approaches to treating damaged ETT with the goal of prolonging their retention on the dental arch and serving as a valuable resource for dental practitioners who face this issue daily.

## 1. Introduction

**Figure 1:**
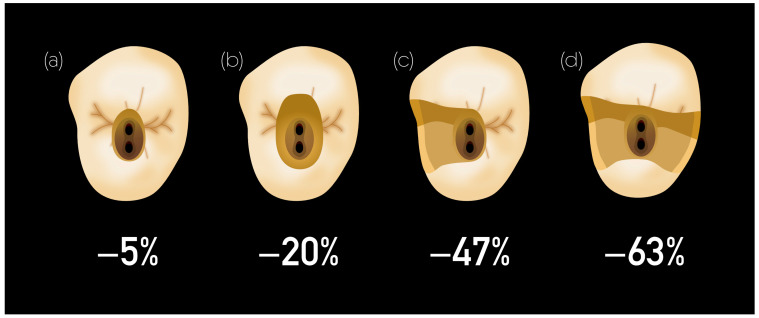
Illustration inspired by the authors of “Reduction in tooth stiffness as a result of endodontic and restorative procedures”, 1989 [6]. (**a**) A conservative access cavity induces a stiffness reduction of 5%; (**b**) an associated occlusal cavity preparation induces a stiffness reduction of 20%; (**c**) an MO or OD cavity preparation induces a stiffness reduction of 47%; (**d**) an MOD cavity preparation induces a stiffness reduction of 63%.

Endodontically treated teeth (ETT) show few but significant differences in mechanical properties compared to vital teeth [1]. Although changes in dentin collagen and moisture content have been described to contribute to the brittleness of ETT over the long term [2,3], it appears that the physical properties of dentin that are clinically relevant remain unchanged by root canal treatments [4,5,6,7]. Conversely, the volumetric loss of the hard tissue caused by decay, preparation, and the whole sequence of root canal treatment play a major role in the risk of fracture [8,9,10]. It has been demonstrated in this context that factors such as the creation of an endodontic access cavity along with the loss of marginal ridges serve as significant static parameters, resulting in maximum tooth fragility [11] (Figure 1).

Moreover, even though periodontal mechanoreceptors remain [12], the intradental receptors that shield the tooth from excessive force are absent in ETT [13], potentially raising the threshold for maximum biting force and modifying proprioception [14,15]. The main reasons for the clinical failure of ETT have been reported as vertical root fractures (12%), cusp fractures (15%), and periodontal issues (40%) [16], underscoring the entire biomechanical problem. Therefore, it is a real challenge for the clinician to restore ETT in a lasting manner as their prognosis not only depends on endodontic and periodontal variables, but also on adequate tooth rehabilitation [17]. Structural resistance relies on the effective retention and adhesive bonding between root dentin, core reconstruction, and final restoration, creating a cohesive and integrated system [18] in which all parts must be considered on their own scale while taking into account the others.

The advent of adhesive dentistry has enabled a minimally invasive approach and opened up new perspectives in the restoration of ETT. The presence of established and reliable adhesive dental techniques has broadened the range of restorative possibilities, and a wide variety of techniques are currently available. It is commonly accepted that the practitioner should aim to conserve as much of the remaining structure as possible, as studies indicate that success is closely linked to both the quantity and quality of the remaining coronal structure [19].

**Figure 2:**
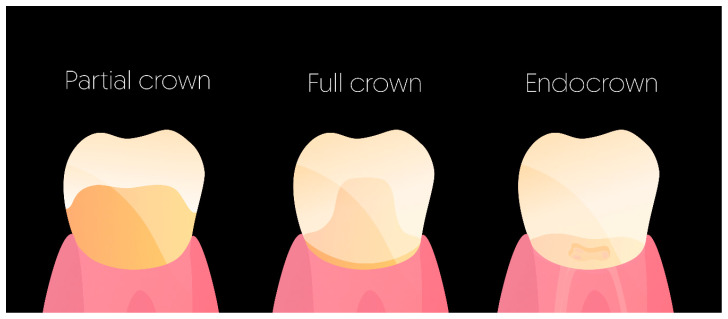
Full-coverage restoration discussed in the present article.

Invariably, teeth undergoing endodontic treatment have lost significant amounts of tooth structure [20]. However, there is no clear definition of “damaged” ETT. Depending on the studies, the authors discuss the presence of ferrule, the number of remaining dentin walls, or the percentage of remaining structure. Recent systematic reviews suggest cuspal coverage when at least one proximal wall is missing, while adopting a conservative approach when designing restorations and utilizing partial coverage restorations, when appropriate, to preserve as much healthy tooth structure as possible [21,22]. In the present study, the authors state that a tooth is damaged as soon as it needs a full cuspal coverage restoration, that is, when the tooth presents an MO/DO cavity with thin axial walls (<2 mm) or any MOD cavity or structure loss beyond an MOD cavity [23]. Hence, partial crowns, full crowns, and endocrowns will be discussed (Figure 2). Conversely, when all four walls are still remaining with a >2 mm thickness, the stiffness reduction provoked by the access cavity is only about 5% [6] (Figure 1). Therefore, only in these cases and in a safe occlusal context can direct adhesive restoration be considered as an alternative to cuspal coverage with a high success rate.

Regardless of the chosen technique, the restoration should be placed as soon as the root canal treatment is completed to maximize the survival rate. Indeed, root-filled posterior teeth restored with cuspal coverage restorations within 4 months of the completion of root canal treatment are three times less likely to be extracted than those restored after 4 months [24].

This article intends to delve into the biomaterial innovations of the past decade and their applications, from crown to root, that offer alternative approaches to the treatment of damaged ETT with the aim of prolonging their retention on the dental arch.

## 2. Methods

A thorough investigation was conducted by reviewing the available literature on the subject, focusing on English-language articles accessible through major search engines (PubMed, Cochrane, Embase, Scopus) and published in prominent indexed journals within the Materials and Dental sector, both with and without impact factors. Priority was given to articles presenting the highest level of evidence available in the literature. The MeSH terms endodontically treated teeth, root filled teeth, damaged, compromised, restoration, crown, endocrown, onlay, partial crown, zirconia, lithium disilicate, composite, CAD-CAM, post, glass-fiber post, cast metal post, cast post, bundled post were used. The recommendations presented in this article are based on articles published over the past 20 years, except for certain conventionally accepted paradigms that are still relevant and may have been introduced in earlier articles without any significant advancements since. The main principles are based on systematic reviews or meta-analyses published in high-impact factor journals. More specific, recent, and less common concepts have been sourced as much as possible from high-impact factor journals whenever feasible. When data were reported from lower impact factor journals, reservations were expressed regarding the conclusions drawn, although their existence in the literature was acknowledged. The initial research included 356 articles after excluding articles older than 20 years (except for the exceptions specified above) and articles with an impact factor < 1 (except for the exceptions specified above). After analyzing either abstract or the entire article, 143 articles were deemed relevant and thus selected.

The results presented in this article were extrapolated from this literature search, with reference to the authors’ clinical experience and biomaterial expertise.

## 3. Results

### 3.1. Choice of Full-Coverage Restoration Material

- Partial crown and veneers

Most ETT have lost a significant amount of substance [25] and need a full-coverage restoration. Nonetheless, tissue preservation must remain at the forefront of the practitioner’s decision-making process as it is considered a critical factor for the long-term clinical prognosis of the restoration [19]. In the posterior sector, when enough substance remains, the bonded partial crown, sometimes called an overlay or occlusal veneer, provides an ideal compromise between cuspal coverage, tissue preservation, and aesthetics. Although few studies have investigated this treatment modality on ETT, the results are promising. In particular, Frankenberger et al. [26] found no considerable difference between partial crown and full crown preparation after in vitro fatigue-loading, regardless of the material, hence recommending the realization of the less invasive option when possible. A more conservative preparation preserves the available enamel, which significantly enhances the predictability of bonded restorations [27]. Regarding the choice of material, the practitioner can opt for machinable composite. Dias et al. [7] reported a 96% success rate and 100% tooth survival rate after 5 years and Chrepa [28] noted a 96.8% success rate and 100% tooth survival after 37 months. Suksawat et al. [29] and Frankenberger et al. [26] reported a promising performance in vitro, while Magne et al. [30] found fewer catastrophic failures with machinable composite than with feldspathic ceramic. Nevertheless, composite exhibits a low elastic modulus and is relatively prone to deformation, placing greater stress on the adhesive joint and leading to a higher degree of marginal leakage [31,32]. Regarding ceramics, feldspathic ceramic should not be the first choice for the realization of partial crowns on posterior ETT, as other materials have shown better performance [30,33,34]. Lithium disilicate and polymer-infiltrated ceramic network (PICN) materials, exhibiting a greater elastic modulus than machinable composites, showed excellent outcomes and can be recommended for partial crowns on ETT [33,35]. Zirconia, on the other hand, was found to present the highest fracture resistance, but also provoked more catastrophic failures [29] and significantly lower marginal adaptation after fatigue loading [26]. Despite well-established zirconia bonding protocols [36], practitioners seem to struggle mastering them, which might impact the quality of the bond. Hybrid materials such as zirconia-reinforced lithium silicate (ZLS) could represent an interesting compromise as it combines the beneficial properties of different materials. Nevertheless, to our knowledge almost no literature mentions its use on ETT, therefore no conclusion can be drawn. Ultimately, Frankenberger [26] stated that the cast-gold partial crown still represents the ultimate tool for safely restoring ETT, although its use is becoming increasingly complicated both economically and technically. In light of this information, lithium disilicate or PICN material should be recommended when restoring ETT with partial crowns in daily practice to optimize bonding, the forces transmitted to the tooth, and the durability of the restoration.

The preparation of partial crowns can sometimes result in an unaesthetic transition between the prosthesis and the tooth that might be discolored following root canal treatment and can be completed using a veneer preparation on the buccal surface to address this issue, leading to a so-called “buccal–occlusal veneer” preparation. This veneer can be prepared using lithium disilicate, and behaves mechanically in the same way as an occlusal veneer [37]. This preparation is more relevant when restoring premolars, which are more visible when smiling. Regarding the cementation, partial crowns must imperatively be bonded with adequate protocols, resulting in superior failure load values compared to full crowns [38].

Regarding the restoration of endodontically treated incisors, very few data are available. Nonetheless, it appears that restoration can be achieved with direct composites up to Class III cavity. However, when a loss of the incisal edge occurs, if needed, a ceramic veneer should be preferred over a crown if enough structure is remaining [39]. This situation still remains rare, as it is challenging to incorporate the access cavity within such restorations, and the significant loss of tooth tissue often results in a considerable reduction in the available bonding surface area [20].

b. Full crown

Crowns are proven to function well as a long-term restoration for ETT [40]. Although full-coverage crowns were widely used in the past and should not be considered as an absolute necessity for restoring ETT, they remain a viable therapeutic option today in many cases of damaged ETT. A recent systematic review and meta-analysis reported a success rate for single crowns of up to 90% after 6 years, regardless of the material used and the placement of the post [41,42]. The success rate seems to increase with the number of remaining walls [43,44], reaching 100% success with four walls remaining. Regarding the material, metal–ceramic crowns have been widely used over recent years, with an estimated 73.33% success rate after 25 years, with dental caries as the main cause of failure [21]. Based on the retrospective data available, gold crowns also demonstrate very high long-term performance [21]. However, primarily for aesthetic reasons, practitioners are now more likely to opt for all-ceramic materials [45]. All-ceramic crowns seem to be an interesting alternative, with sufficient strength to withstand functional forces, along with aesthetic benefits. It should be noted that studies addressing the success rates of different materials for crowns on ETT are very limited. In the absence of data, various systematic reviews on the restoration of ETT readily extrapolate findings from pools where both vital teeth and ETT are mixed. Recent systematic reviews concluded that all-ceramic crowns made of leucite, lithium-disilicate-reinforced glass ceramic, or alumina-based oxide ceramics could be suggested as a substitute for gold-based metal ceramic crowns for both anterior and posterior teeth. Feldspathic and silica-based ceramics were only deemed suitable and safe for anterior restorations [21]. Layered zirconia-based crowns were found to be inferior due to retention loss and ceramic veneering fracture [17,21]. Even fewer studies are available regarding the use of monolithic zirconia crowns on ETT. Frankenberger et al. [26] showed that monolithic zirconia full crowns had the highest post-fatigue fracture resistance in vitro on ETT. Monolithic zirconia allows for very thin preparation because of its mechanical properties and good machinability. These minimal preparations allow for the preservation of a greater amount of tooth structure, which is of crucial importance for ETT. Considering this information, monolithic zirconia crowns should certainly be considered an option by the practitioner even though more studies are needed. Wang et al. [46], in their recent meta-analysis combining in vitro studies and clinical trials, concluded that zirconia was the best material for full crowns on the basis of the fracture resistance and mode. Nevertheless, this study included vital teeth and was, therefore, not specific to ETT. Although it is mechanically very strong, its highly opaque appearance restricts its aesthetic use in the anterior region. For a more natural aspect, lithium disilicate [47] and zirconia are preferred in anterior teeth in layered forms to enhance translucency.

The long-term survival of composite crowns has not been evaluated in terms of ETT. Although these materials have evolved, concerns persist regarding their long-term wear and fracture resistance, as well as marginal discoloration. Specifically, there is apprehension regarding their strength in regions subject to both high-functional and nonfunctional stresses. Indeed, the time-dependent patterns of marginal breakdown raise concerns about the long-term stability of the restoration [22]. In anterior teeth being subjected to a particular mechanical context, especially concerning high shear forces [21], no evidence has so far recommended the use of machinable composites.

c. Endocrowns

Endocrowns are described as adhesive monolithic restorations anchored in the pulp chamber, exploiting the micromechanical retention properties of the pulp–chamber walls [32], and have appeared with the progress in the development of adhesive techniques. The difference with a traditional full crown lies in the fact that no additional restorations (such as a post or composite build-up) are associated with it, reducing the number of clinical steps and preserving the maximum amount of sound tooth tissue. The core and the crown are assembled in one single component [48,49]. Given that the stiffness mismatch between dentin, luting cement, and the restorative system can affect stress distribution—with more interfaces between different materials, leading to poorer stress distribution—the monoblock nature of endocrowns can better support stress loading compared to the multi-interface nature of conventional restorations [50]. Despite the growing popularity of endocrown restorations, the question remains as to whether clinicians should opt for endocrowns over traditional treatments involving intraradicular posts. Clinical evidence in the literature is still limited, with existing studies having relatively short follow-up periods, not exceeding 3 years. Nonetheless, there are a fair number of in vitro studies available that report on the fracture strength of endocrowns [42]. Recent systematic reviews and meta-analyses reported equivalent or sometimes higher success rates of endocrowns compared to post-retained crowns on molars [32,42]. Therefore, although more clinical data on endocrowns are generally needed, it can be concluded that it might be a suitable alternative for restoring damaged root-filled molars, provided the adhesive luting procedure is performed correctly [21].

Despite a lack of evidence, it has been noted that endocrowns fail more frequently when placed on premolars and incisors, likely due to their smaller adhesion area and greater crown height compared to molars [32,42]. Additionally, these teeth are subjected to more non-axial forces than molars, which may also impact fracture resistance [27]. Therefore, endocrowns cannot be currently recommended for use on incisors and premolars.

Regarding the choice of materials, machinable composites have advantageous characteristics for endocrown fabrication due to their modulus of elasticity, which closely matches that of dentin. This similarity helps limit irreparable fractures while maintaining high fracture resistance. However, once again, a lower elastic modulus increases the stress at the interface, which can lead to risks of debonding and prosthesis detachment. The same concerns arise with PICN [47,48]. Since debonding has been reported as the most common cause of failure by Ploumaki et al. [41], more so than the risk of fracture, materials with the highest adhesion values, such as lithium disilicate, are the best choice [32]. The aesthetic properties of lithium disilicate surpass those of composite resin, and it also ages better and has lower plaque retention [51].

There is a lack of evidence regarding the use of zirconia in this indication. Even though it appears promising due to its excellent mechanical properties, it has exhibited the highest rate of catastrophic failure among other materials [32,52,53] and should not be recommended as a first-intention material for endocrowns.

### 3.2. Post or No Post?

Root canal posts have been recommended for anchorage and the retention of the core build-up and final coronal restoration. They were generally recommended when minimal or no coronal tooth structure was available for anti-rotational features and bonding [54]. However, with advancements in dentin bonding and the ability to forego retentive preparations, the relevance and necessity of their use are currently being questioned. Despite the clinical success achieved with the use of intraradicular posts, the main disadvantage is the additional removal of sound tissue in order to fit the post into the root canal. Furthermore, this procedure has been shown to impact the overall biomechanical behavior of the restored teeth. Therefore, the extra retention provided by a post must be weighed against the loss of healthy tooth tissue, which inevitably weakens the tooth [42]. Although there have been attempts to classify the indications for posts by considering important factors such as crown height, wall thickness, circumferential integrity, and the diameter and shape of the canal, there is still no general consensus on when post placement is necessary [55]. Nevertheless, certain trends seem to be now emerging.

**Figure 3:**
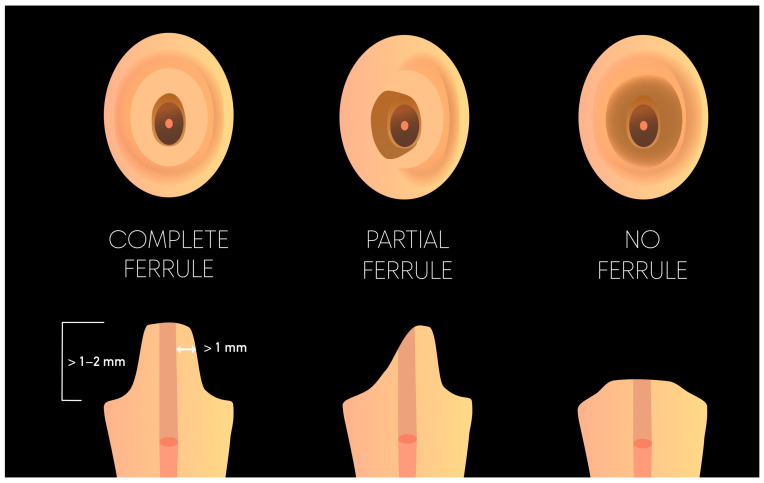
Illustration of the ferrule effect.

In the presence of a ferrule, both in vitro and in vivo studies strongly suggest that posts are unnecessary for restoring ETT [10,55,56,57,58]. A ferrule is defined as the remaining natural tooth structure between the apical extension of the tooth/core junction and the crown preparation margin [59]. Clinically, it is widely accepted that walls are considered “too thin” if they are less than 1 mm thick, meaning the minimal ferrule height is only beneficial if the remaining dentin is at least 1 mm thick [60]. The longer the ferrule, the better, with some studies suggesting a minimum height of 1 mm [61,62,63] (Figure 3). The significance of the ferrule in prolonging the lifespan of restored ETT has been widely studied, with numerous studies demonstrating its beneficial effect on fracture resistance [64]. Therefore, a circumferential ferrule (CF) can be considered as the first ideal solution for restoration of ETT and should be sought whenever possible [65]. Nevertheless, it is not possible to secure/provide CF in all clinical cases. Therefore, the clinical decision must balance the benefits and risks of achieving an “all-around” uniform ferrule. The potential complications of a crown-lengthening procedure include damage to adjacent teeth, the reduction of attached gingiva width, tooth sensitivity, and the risk of postoperative tooth recession [66]. These complications should be weighed against the biomechanical risks associated with a crown lacking a complete ferrule. Indeed, an incomplete ferrule has been described as an appropriate alternative option in ETT [59], whereas no ferrule at all undeniably affects tooth survival in the long term [67].

Studies indicate that posts are not required to retain crowns or endocrowns and may even be linked to higher rates of catastrophic failures. When no ferrule can be obtained, the placement of a post still seems beneficial on anterior teeth and premolars due to the higher risk of mechanical failure in this region [17,68,69,70]. Regarding molars, which have a larger bonding surface due to the size of their pulpal floor, the placement of a post is not justified, even in the absence of coronal walls [17,19].

- No post required: Choice of material for composite build-up

**Figure 4:**
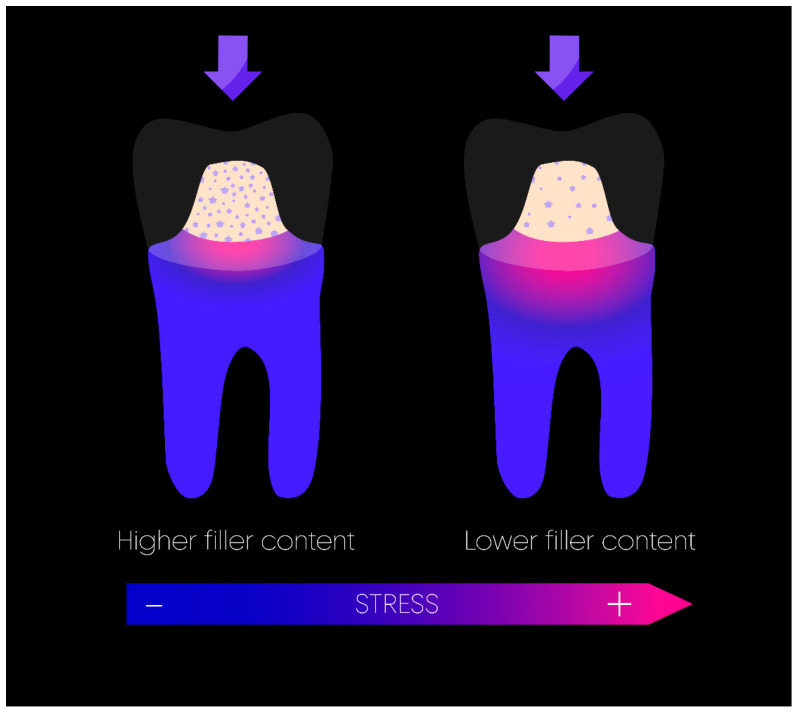
Stress distribution based on the amount of filler in the composite build-up. An increased amount of filler results in less stress at the interface.

If the practitioner chooses not to use a post, two solutions exist for the restoration of the tooth core: the placement of an endocrown or the insertion of an intermediate plastic core material onto which the full coverage restoration will be placed. It was demonstrated that the performance of all-ceramic crowns is influenced by the elastic modulus of the core buildup [55]. In this indication, composite offers the best mechanical properties when compared to IRM [29] or glass ionomer cement (GIC) [71]. Nevertheless, it has been shown that the polymerization of the resin matrix could impact the stability of the restoration. Polymerization shrinkage, depending on the concentration, type, and flexibility of the reacting groups, can lead to varying degrees of shrinkage stress as monomer molecules transition into a polymer network. This stress may lead to marginal deficiencies, enamel fractures, cusp movements, and cracked cusps, which, in turn, could cause microleakage and secondary caries [72]. For ETT in particular, excessive polymerization shrinkage on a mechanically weakened structure can only be unfavorable. In this context, “bulk-fill” composites have been developed to reduce cuspal strain and stress after polymerization [73]. Martins et al. [74] showed higher stress levels in the incrementally filled conventional restorations compared to bulk-filled ones, while the fracture loads were not statistically significantly different. These materials have been proven effective in both laboratory studies and clinical settings, exhibiting reliability equal to or greater than conventional composites [75,76]. Even in cavities with high C-factors, such as those found in ETT with minimal loss of coronal structure, bulk-fill composites have demonstrated strong adhesion [40]. A recent meta-analysis suggested that a composite core build-up with a higher filler content tended to improve the fracture resistance of the endodontically treated teeth in comparison with conventional composite resins in vitro [77] (Figure 4). Therefore, a high-filled bulk-fill composite can be recommended for composite build-up in ETT. Nevertheless, the lower viscosity of bulk-fill flowable composite allows an easier application inside narrower spaces, such as an access cavity, and remains interesting in this indication. Moreover, Oliveira et al. found no difference in the fracture strength between flowable bulk-fill and classic bulk-fill composites [76]. Eventually, dual-core composite resins, which also exhibit a higher filler composition, showed similar performance and can be recommended [76,78].

**Figure 5:**
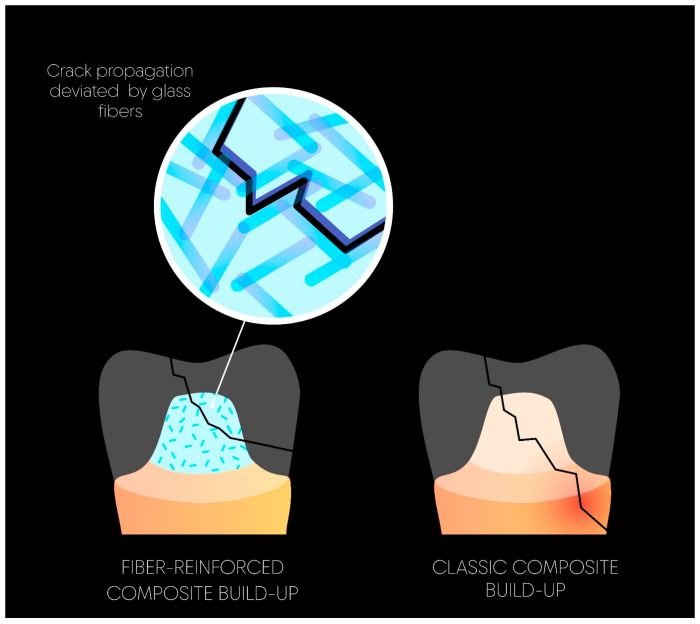
Crack propagation is guided by the glass fibers, resulting in tooth protection and fewer catastrophic failures.

Even more recently, advanced short-fiber-reinforced composite (SFRC) materials have been described as providing structural and chemical reinforcement to weaker teeth [79] and even having the potential to prevent fractures in ETT [80]. Indeed, the structure and orientation of the short fibers, in combination with the composite resin matrix, may allow for more constrained crack propagation following force application [81]. Hence, the material acts like a fuse and can stop cracks or propagate the fracture only within itself, preserving the tooth from catastrophic failures more efficiently [82] (Figure 5). Due to their enhanced physical and mechanical properties, SFRCs are recommended for the biomimetic replacement of dentin in larger cavities and ETT, as they enhance mechanical retention, inhibit fracture propagation, and establish robust chemical bonding between glass fibers and the resin matrix. Selvaraj et al. [79], in their systematic review in 2023, showed that substituting conventional hybrid composites with fiber-reinforced composites enhances the fracture resistance of ETT. Indeed, fifteen studies reported consistent findings that the fracture resistance of fiber-reinforced composites is higher than for conventional hybrid composites. The data are contradictory regarding the polymerization shrinkage of short-fiber-reinforced composites (SFRCs), with some authors describing lower shrinkage stress for SFRCs compared with bulk-filled types and others finding the contrary [82]. Both bulk-fill and fiber-reinforced composite materials are currently available on the market. Therefore, the combination of these two properties seems very promising for core restorations. Fiber-reinforced composite should always be covered with a classic composite [83] in order to prevent hydrolysis between the fibers and the matrix.

For composite build-ups in ETT without post placement, high success rates were found after up to 10 years [84], regardless of tooth type or the number of restored tooth surfaces, along with improved fracture resistance [77]. Finally, to obtain a lower failure rate, the volume of the coverage/restoration material should be maximized at the expense of the core build-up volume, and the build-up should be arithmetically uniform; that is, its height should be proportional to the adhesive surface available [85].

b. Post required: which one is more favorable?

Clinicians have many choices regarding the choice of post material: rigid, like titanium, stainless steel, gold alloys, or zirconia; or flexible, like carbon, glass, or quartz fiber posts [21].

**Figure 6:**
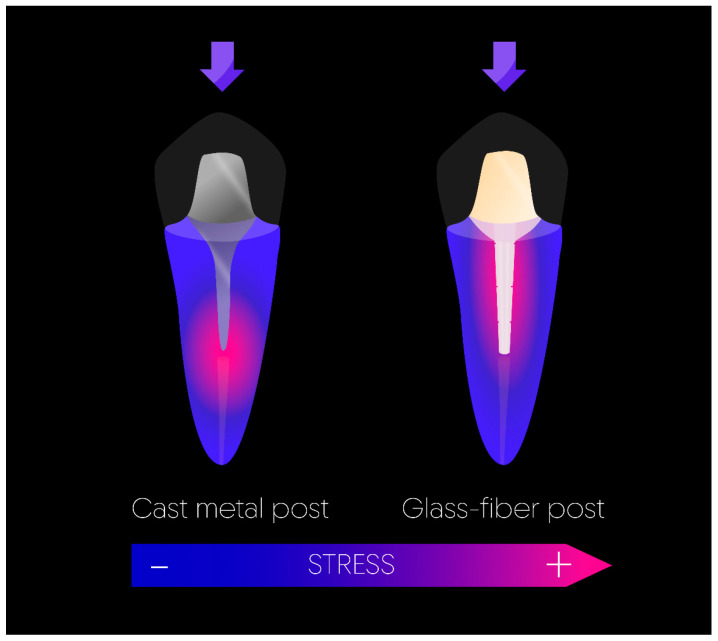
Stress distribution based on the type of post. The cast metal post concentrates the stress at its base, while the distribution is more uniform along the root with the glass-fiber post.

Gold alloy or cobalt–chromium-based cast posts and cores have been utilized for many years in the restoration of root-filled teeth, with clinical trials indicating high success rates ranging from 84% to 94% after 10 years [86,87,88]. Nevertheless, they are characterized by their high compressive strength and modulus of elasticity, having the drawback of transferring masticatory forces to the tooth structure, which may result in irreversible fracture [19]. In recent years, prefabricated fiber-reinforced posts have become increasingly favored for clinical use over cast posts. This shift in preference could be attributed to improved aesthetics and reduced treatment times. Furthermore, the similarity in the elastic modulus between fiber posts and dentin may contribute to a decreased risk of root fractures [89,90]. Given that the mechanical properties of the entire system, encompassing the post, cement, and dentine, should be uniform, utilizing fiber posts cemented and restored with composite resin material is likely to result in satisfactory performance [91] (Figure 6). Moreover, a meta-analysis of thirteen in vitro studies evaluated the fracture resistance of ETT restored with cast posts versus glass-fiber posts and concluded that the cast post group exhibited significantly higher fracture resistance compared to the glass-fiber post group [92]. In vitro studies are interesting to simulate clinical circumstances to predict behaviors, but clinical trials remain the ultimate instrument in restorative dentistry [16,93,94,95]. Yet, recent clinical studies failed to demonstrate a difference in the failure rate between cast-post and glass-fiber posts; therefore, the most recent meta-analysis concluded that both were equivalent and recommended following the preference of the professional or individual characteristics of the patient [96,97]. The fact that using glass-fiber posts incurs a lower annual cost compared to using cast posts could be taken into account in the decision [98]. When a metal post is selected, the shape of the post and the choice of metal material should be carefully considered, as each combination could result in a more or less favorable stress distribution [99].

Whether cast or glass-fiber posts are used, the insertion of a post necessarily involves the removal of healthy tooth substance, mechanically weakening the tooth. As mentioned earlier, a key factor for the long-term survival of root-filled teeth is the amount of remaining coronal tooth structure. Therefore, the removal of tooth structure during post space preparation should be avoided [21]. In this context, the recent development of multi-fiber-reinforced composite posts (mFRCPs), also called bundled glass-fiber-reinforced posts or bundled posts (BPs), is promising. The use of BPs in the root canal space does not necessitate post space preparation as it is based on a bundle of fibers that are bonded directly to the root canal [100]. To our knowledge, only in vitro studies regarding this technique exist. It has been reported that BPs improve the resistance and stress distribution compared to single fiber posts in incisors [101]. The same study showed that BPs combined with single fiber posts improved the results on immature incisors. Most of the studies reported that the utilization of multiple posts in the weakened root canal provided better fracture resistance and stress distribution in both anterior and posterior regions [101,102,103,104,105]. Sturm et al. [106] even found that teeth restored with BP showed higher fracture resistance than those restored without posts, suggesting a reinforcement of ETT. The bond strength to the canal was found to be comparable to or higher than single fiber posts [100,107]. Sturm et al. [106] found a difference in the C-factor by a factor of 2.5–5.6 between single fiber posts and BPs, depending on the individual tooth parameters of the compared samples. This suggests a favorable shrinkage strain and stress distribution during the hardening of the composite within samples of adhesively luted bundled fiber posts.

It is worth noting that some other studies did not find any difference in the fracture resistance between BPs and single fiber posts [108,109]. Hence, as the bundled fiber post does not necessitate tooth preparation, it should be preferred over single fiber posts.

**Figure 7:**
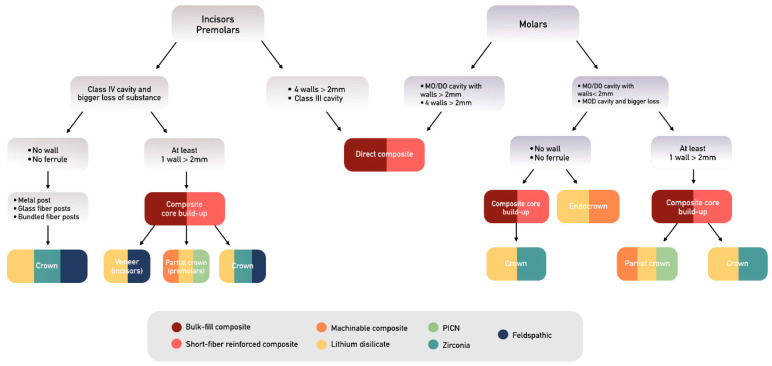
Decision tree for restoring ETT.

The question of reintervention arises with this kind of post, but some authors suggested adding a gutta-percha point in the center of the fiber bundle, which, in case of root reinfection, enables easier reintervention than when a metal or single fiber post is used [104]. In any case, considering that the placement of a post or a composite build-up complicates the possibility of reintervention, endodontic microsurgery, which is now widely adopted and has excellent success rates [110], should be considered each time it is possible in these situations. Although very promising, more evidence is needed to systematize their utilization (Figure 7).

It has been reported that a post should have a functionally graded stiffness that decreases from the coronal part to the apical end to optimize stress distribution [111], which could be accomplished through an inhomogeneous post design with hybrid materials [112]. This appears to be a promising direction to investigate.

c. Post cementation

Achieving reliable intraradicular dentine adhesion remains a clinical challenge due to limited access and visibility, light inaccessibility, and a reduced number of dentinal tubules in the apical third. This is further complicated by the presence of irregular secondary dentine and other structures, which increases the risk of the system debonding over long periods in the oral environment [113]. Resin luting cements are preferred for post cementation because they provide satisfactory retention and resistance against post fracture [57]. However, the anatomical and histological characteristics of the root canal can influence the adhesion of luting resin cement, leading to variations in dentin bonding across different areas of the same tooth. In particular, bond strength was found to be significantly higher in the cervical third by many studies [114,115,116,117], raising the question of the appropriate post length. Mobilio et al. reported that unless the intracanal post length is less than one-third of the root length, the impact on the fracture resistance of the treated teeth may not be significant, provided that the proper cementation protocol is adhered to [118]. Finally, Mastsumoto et al. [119] reported that only the first 2 mm of the coronal part of the root canal offers interesting bond strength. Regarding this information, post length should not exceed one-third of the root length in the case of an anterior tooth or premolar with no ferrule, and in all other cases, composite build-up should be preferred, with potential extra retention brought by the bonding composite in the first 2 mm of the canal.

Regarding the types of resin luting cements available, although adhesive resin luting cements with associated etch-and-rinse adhesives have traditionally been proposed, advances in adhesive technology have led to simplified protocols using self-etch or universal adhesives with adhesive resin luting cements, but also directly self-adhesive resin cement without any previous surface treatment [120,121]. These innovations have shortened chair time and streamlined clinical procedures. Moreover, the use of self-adhesive resin cement eliminates the challenging task of applying and rinsing phosphoric acid in the apical area of the prepared canal, resulting in a more predictable and less technique-sensitive procedure [122,123]. De Morais et al. [120], in their 2023 narrative review, reported that most studies demonstrated significantly higher bond strength values for self-adhesive luting cement, while some others have shown comparable performance among the various adhesive systems available. Angnanon et al. [124], in their 2023 network meta-analysis, found similar results, with self-adhesive cement exhibiting significantly better effectiveness than resin luting cement in long-term aging conditions. Resin cement associated with self-etch adhesives showed superior performance in the short term but yielded lower bond strength after aging.

To address the problem of light inaccessibility in the canals, dual-cure resin luting cement should be used, although it has been reported that when a dual-cure adhesive is only chemically polymerized, the strength of the bond to root canal dentin is lower compared with light-activated polymerization [125]. More recently, universal resin luting cements appeared as a new simplified solution and a great alternative in this indication. Universal self-adhesive luting resin cement is touch-cure-activated by an associated primer or adhesive, eliminating the need for photopolymerization. This alternative has been described to be more efficient than dual-cure resin luting cement for some formulations and seems very promising [126].

In terms of procedures to be carried out on the intraradicular dentin before conducting the adhesive procedure, the deleterious effect of endodontic procedures on the bond strengths, such as irrigation with high-concentration sodium hypochlorite or the use of zinc oxide–eugenol cements interacting with the polymerization reaction, is well documented [127,128,129]. When a self-adhesive strategy is employed, the use of ethanol for the decontamination of intracanal dentin appears to be the best solution. When more complex systems are used in combination with adhesives used in etch-and-rinse mode, decontamination with low-concentration hypochlorite appears to be the best procedure to implement before the specific adhesive protocol. However, ethanol also appears to be efficient and has been described in more studies [127]. Therefore, the systematic use of ethanol rinsing before intracanal bonding seems to be the most advisable procedure.

Regarding the cleaning and conditioning of contaminated core build-up material before adhesive bonding, cleaning with pumice or air abrasion seemed superior to using polishing powder or phosphoric acid [130]. Silane was a less effective conditioning agent compared to composite or dentin primers. Ideally, after contamination, bonding surfaces should be cleaned with a pumice suspension and conditioned with a dentin adhesive. Alternatively, these surfaces can be cleaned and conditioned using air abrasion with alumina particles and a composite resin primer.

### 3.3. Deep Margin Elevation

The significant loss of substance in ETT can quickly lead to subgingival defects, resulting in difficulties in managing moisture and contamination to achieve a high-quality restoration. To preserve and restore teeth with subgingival defects, various treatment options are available, including surgical extrusion, also referred to as intra-alveolar transplantation or intentional replantation; surgical crown lengthening; and orthodontic forced eruption [131,132,133,134]. Although effective, some of these techniques are invasive, and others significantly extend clinical times. In this context, deep margin elevation (DME) emerged, which consists of direct restoration to reposition the cervical margin to a supragingival position, making it easier to isolate the working area with a rubber dam, take conventional or digital impressions, bond an indirect restoration, and remove any excess luting material [135]. As root canal treatment already requires significant chair time, reducing the number of visits for restoring ETT might be desirable. Nonetheless, if the defect involves the buccal or lingual part of the tooth, surgical crown lengthening or surgical extrusion should be considered [136]. The remaining ferrule could also guide the choice of whether to opt for DME or crown lengthening in ETT. Indeed, Falahchai et al. recently demonstrated that teeth that exhibited a partial ferrule benefited in terms of fracture resistance from DME rather than crown lengthening [137]. The difference in fracture resistance was not significant between DME and crown lengthening on ETT with a complete ferrule, but the frequency of unfavorable fractures was clearly higher in the crown lengthening group, guiding the choice toward DME. Even if most of studies are not specific to ETT, DME has been described as a very effective technique with a more than 95% survival rate after 12 years [138]. Mechanically, this technique did not weaken the tooth [139,140,141], which is of crucial importance in ETT. From a histological perspective, no connective attachment can be achieved with the material. Instead, DME results in the formation of a different biological width, primarily consisting of a long junctional epithelium and a slight connective attachment to the dentin below the material. However, this condition appears healthy and well-tolerated by the body [142] even though some authors described an increased inflammation [143]. Some studies were exclusively conducted on ETT, notably Farouk et al., who conducted a randomized control trial and concluded that DME was clinically successful with favorable biologic responses [144]. In addition, Ilgentstein et al. found that it had no impact on either the marginal integrity or the fracture behavior of root-canal-treated mandibular molars restored with ceramic onlays in vitro [145].

Regarding the choice of material, there is no consensus on which is the more suitable [141], but composite appears to be a good choice, whereas glass ionomer (GI) should not be recommended as it has shown many catastrophic failures [146] that may be attributed to the chemical bond of GI to dentin. Self-adhesive resin-based luting materials are also not suitable for DME [141]. DME often involves narrow spaces; therefore, flowable material can be an interesting option to enable full access of the material to the entire cavity. More recently, highly filled flowable resin composites showed promising results in finite element analyses when applied to deep margin elevation from an interfacial mechanical point of view [147], combining flowability and good mechanical properties.

### 3.4. Limits and Future Directions

This article does not constitute a systematic review. Therefore one notable limit in this study is the subjective nature of the article selection process, which was influenced by the authors’ own experience. The criteria for including or excluding articles were based on the impact factor level of the journals, and the author’s judgment which inherently introduces a level of subjectivity. While the authors aimed to include the most pertinent and high-quality research, the process inevitably reflects their individual biases and professional background and this should be considered when interpreting the article.

Current methods for restoring endodontically treated teeth focus on highly reliable adhesive procedures and high-strength ceramics. Future direction in this field could explore hybrid materials—that combine the best properties of different materials—whether it concerns posts or coronal restorations. Research on posts should focus on techniques that would not necessitate an additional canal preparation. More randomized clinical trials should be conducted to assess the long-term survival of root-filled teeth restored using minimal intervention endodontic-restorative concepts and techniques. Regarding reintervention, authors truly believe that micro-endodontic surgery should be sought whenever faisible and when not, cases with posts to remove should be adressed to a endodontics specialist.

## 4. Conclusions

The survival rate of teeth and restorations after root canal treatment is influenced by numerous variables, and the evidence base for restoring ETT remains complex and unclear. Hence, it is difficult to evaluate the impact of each factor on tooth or restoration survival individually in a randomized clinical trial, given the challenge of standardizing all other variables. However, trends seem to progressively emerge in various reviews and meta-analyses, providing practitioners strong scientific data to support their practice.

Future research should focus on conducting randomized clinical trials to assess the long-term survival of root-filled teeth restored using minimal intervention endodontic-restorative concepts and techniques.

## Data Availability

The data that support the findings of this study are available from the corresponding author, [E.C.], upon reasonable request.

## References

[1] Sedgley C.M., Messer H.H. (1992). Are endodontically treated teeth more brittle?. J. Endod..

[2] Soares C.J., Santana F.R., Silva N.R., Preira J.C., Pereira C.A. (2007). Influence of the Endodontic Treatment on Mechanical Properties of Root Dentin. J. Endod..

[3] Tang W., Wu Y., Smales R.J. (2010). Identifying and Reducing Risks for Potential Fractures in Endodontically Treated Teeth. J. Endod..

[4] Papa J., Cain C., Messer H.H. (1994). Moisture content of vital vs. endodontically treated teeth. Endod. Dent. Traumatol..

[5] Fusayama T., Maeda T. (1969). Effect of pulpectomy on dentin hardness. J. Dent. Res..

[6] Reeh E.S., Messer H.H., Douglas W.H. (1989). Reduction in tooth stiffness as a result of endodontic and restorative procedures. J. Endod..

[7] Dias M.C.R., Martins J.N.R., Chen A., Quaresma S.A., Lúıs H., Caramês J. (2018). Prognosis of Indirect Composite Resin Cuspal Coverage on Endodontically Treated Premolars and Molars: An In Vivo Prospective Study. J. Prosthodont..

[8] Dietschi D., Duc O., Krejci I., Sadan A. (2007). Biomechanical considerations for the restoration of endodontically treated teeth: A systematic review of the literature—Part 1. Composition and micro- and macrostructure alterations. Quintessence Int..

[9] Dietschi D., Duc O., Krejci I., Sadan A. (2008). Biomechanical considerations for the restoration of endodontically treated teeth: A systematic review of the literature, Part II (Evaluation of fatigue behavior, interfaces, and in vivo studies). Quintessence Int..

[10] Reeh E.S., Douglas W.H., Messer H.H. (1989). Stiffness of endodontically-treated teeth related to restoration technique. J. Dent. Res..

[11] Linn J., Messer H.H. (1994). Effect of restorative procedures on the strength of endodontically treated molars. J. Endod..

[12] Piancino M.G., Isola G., Cannavale R., Cutroneo G., Vermiglio G., Bracco P., Anastasi G.P. (2017). From periodontal mechanoreceptors to chewing motor control: A systematic review. Arch. Oral Biol..

[13] Dong W.K., Chudler E.H., Martin R.F. (1985). Physiological properties of intradental mechanoreceptors. Brain Res..

[14] Awawdeh L., Hemaidat K., Al-Omari W. (2017). Higher Maximal Occlusal Bite Force in Endodontically Treated Teeth Versus Vital Contralateral Counterparts. J. Endod..

[15] Loewenstein W.R., Rathkamp R. (1955). A study on the pressoreceptive sensibility of the tooth. J. Dent. Res..

[16] Touré B., Faye B., Kane A.W., Lo C.M., Niang B., Boucher Y. (2011). Analysis of reasons for extraction of endodontically treated teeth: A prospective study. J. Endod..

[17] Mannocci F., Bitter K., Sauro S., Ferrari P., Austin R., Bhuva B. (2022). Present status and future directions: The restoration of root filled teeth. Int. Endod. J..

[18] Soares C.J., Rodrigues M.d.P., Faria-e-Silva A.L., Santos-Filho P.C.F., Veríssimo C., Kim H.C., Versluis A. (2018). How biomechanics can affect the endodontic treated teeth and their restorative procedures?. Braz. Oral Res..

[19] de Matos L.M.R., Silva M.L., Cordeiro T.O., Cardoso S.d.A.M., Campos D.e.S., de Muniz I.A.F., Barros S.A.d.L., Seraidarian P.I. (2024). Clinical and laboratorial performance of rehabilitation of endodontically treated teeth: A systematic review. J. Esthet. Restor. Dent..

[20] Mannocci F., Cowie J. (2014). Restoration of endodontically treated teeth. Br. Dent. J..

[21] Bhuva B., Giovarruscio M., Rahim N., Bitter K., Mannocci F. (2021). The restoration of root filled teeth: A review of the clinical literature. Int. Endod. J..

[22] Mannocci F., Bhuva B., Roig M., Zarow M., Bitter K. (2021). European Society of Endodontology position statement: The restoration of root filled teeth. Int. Endod. J..

[23] Abu-Awwad M. (2019). Dentists’ decisions regarding the need for cuspal coverage for endodontically treated and vital posterior teeth. Clin. Exp. Dent. Res..

[24] Pratt I., Aminoshariae A., Montagnese T.A., Williams K.A., Khalighinejad N., Mickel A. (2016). Eight-Year Retrospective Study of the Critical Time Lapse between Root Canal Completion and Crown Placement: Its Influence on the Survival of Endodontically Treated Teeth. J. Endod..

[25] Bandlish R.B., McDonald A.V., Setchell D.J. (2006). Assessment of the amount of remaining coronal dentine in root-treated teeth. J. Dent..

[26] Frankenberger R., Winter J., Dudek M.C., Naumann M., Amend S., Braun A., Krämer N., Roggendorf M.J. (2021). Post-Fatigue Fracture and Marginal Behavior of Endodontically Treated Teeth: Partial Crown vs. Full Crown vs. Endocrown vs. Fiber-Reinforced Resin Composite. Materials.

[27] Ferrari M., Ferrari Cagidiaco E., Pontoriero D.I.K., Ercoli C., Chochlidakis K. (2022). Survival Rates of Endodontically Treated Posterior Teeth Restored with All-Ceramic Partial-Coverage Crowns: When Systematic Review Fails. Int. J. Environ. Res. Public Health.

[28] Chrepa V., Konstantinidis I., Kotsakis G.A., Mitsias M.E. (2014). The survival of indirect composite resin onlays for the restoration of root filled teeth: A retrospective medium-term study. Int. Endod. J..

[29] Suksawat N., Angwaravong O., Angwarawong T. (2024). Fracture resistance and fracture modes in endodontically treated maxillary premolars restored using different CAD-CAM onlays. J. Prosthodont. Res..

[30] Magne P., Knezevic A. (2009). Influence of overlay restorative materials and load cusps on the fatigue resistance of endodontically treated molars: Quintessence International. Quintessence Int..

[31] Sevimli G., Cengiz S., Oruc M.S. (2015). Endocrowns: Review. J. Istanb. Univ. Fac. Dent..

[32] Govare N., Contrepois M. (2020). Endocrowns: A systematic review. J. Prosthet. Dent..

[33] Lu T., Peng L., Xiong F., Lin X.Y., Zhang P., Lin Z.T., Wu B.-L. (2018). A 3-year clinical evaluation of endodontically treated posterior teeth restored with two different materials using the CEREC AC chair-side system. J. Prosthet. Dent..

[34] Scherrer S.S., de Rijk W.G. (1993). The fracture resistance of all-ceramic crowns on supporting structures with different elastic moduli: International Journal of Prosthodontics. Int. J. Prosthodont..

[35] Xiao W., Chen C., Yang T., Zhu Z. (2020). Influence of Different Marginal Forms on Endodontically Treated Posterior Teeth Restored with Lithium Disilicate Glass-Ceramic Onlays: Two-Year Follow-up. Int. J. Prosthodont..

[36] Zarone F., Di Mauro M.I., Ausiello P., Ruggiero G., Sorrentino R. (2019). Current status on lithium disilicate and zirconia: A narrative review. BMC Oral Health.

[37] Huang X.Q., Hong N.R., Zou L.Y., Wu S.Y., Li Y. (2020). Estimation of stress distribution and risk of failure for maxillary premolar restored by occlusal veneer with different CAD/CAM materials and preparation designs. Clin. Oral Investig..

[38] Spitznagel F.A., Prott L.S., Hoppe J.S., Manitckaia T., Blatz M.B., Zhang Y., Langner R., Gierthmuehlen P.C. (2024). Minimally invasive CAD/CAM lithium disilicate partial-coverage restorations show superior in-vitro fatigue performance than single crowns. J. Esthet. Restor. Dent..

[39] von Stein-Lausnitz M., Mehnert A., Bruhnke M., Sterzenbach G., Rosentritt M., Spies B.C., Bitter K., Naumann M. (2018). Direct or Indirect Restoration of Endodontically Treated Maxillary Central Incisors with Class III Defects? Composite vs. Veneer or Crown Restoration. J. Adhes. Dent..

[40] Atlas A., Grandini S., Martignoni M. (2019). Evidence-based treatment planning for the restoration of endodontically treated single teeth: Importance of coronal seal, post vs. no post, and indirect vs. direct restoration. Quintessence Int..

[41] Ploumaki A., Bilkhair A., Tuna T., Stampf S., Strub J.R. (2013). Success rates of prosthetic restorations on endodontically treated teeth; a systematic review after 6 years. J. Oral Rehabil..

[42] Sedrez-Porto J.A., da Rosa W.L.D.O., Da Silva A.F., Münchow E.A., Pereira-Cenci T. (2016). Endocrown restorations: A systematic review and meta-analysis. J. Dent..

[43] Afrashtehfar K.I., Ahmadi M., Emami E., Abi-Nader S., Tamimi F. (2017). Failure of single-unit restorations on root filled posterior teeth: A systematic review. Int. Endod. J..

[44] AlSaleh E., Dutta A., Dummer P.M.H., Farnell D.J.J., Vianna M.E. (2021). Influence of remaining axial walls on of root filled teeth restored with a single crown and adhesively bonded fibre post: A systematic review and meta-analysis. J. Dent..

[45] Edelhoff D., Brix O. (2011). All-ceramic restorations in different indications: A case series. J. Am. Dent. Assoc..

[46] Wang B., Fan J., Wang L., Xu B., Wang L., Chai L. (2022). Onlays/partial crowns versus full crowns in restoring posterior teeth: A systematic review and meta-analysis. Head. Face Med..

[47] Fabbri G., Zarone F., Dellificorelli G., Cannistraro G., De Lorenzi M., Mosca A., Leone R., Sorrentino R. (2024). A 13- to 17-year Retrospective Evaluation of the Clinical Performances of Anterior and Posterior Lithium Disilicate Restorations onto Teeth and Implants. Int. J. Periodontics Restor. Dent..

[48] Otto T. (2004). Computer-aided direct all-ceramic crowns: Preliminary 1-year results of a prospective clinical study. Int. J. Periodontics Restor. Dent..

[49] Zarone F., Sorrentino R., Apicella D., Valentino B., Ferrari M., Aversa R., Apicella A. (2006). Evaluation of the biomechanical behavior of maxillary central incisors restored by means of endocrowns compared to a natural tooth: A 3D static linear finite elements analysis. Dent. Mater..

[50] Tay F.R., Pashley D.H. (2007). Monoblocks in root canals: A hypothetical or a tangible goal. J. Endod..

[51] Lempel E., Gyulai S., Lovász B.V., Jeges S., Szalma J. (2023). Clinical evaluation of lithium disilicate versus indirect resin composite partial posterior restorations—A 7.8-year retrospective study. Dent. Mater..

[52] Ciobanu P., Manziuc M.M., Buduru S.D., Dudea D. (2023). Endocrowns—A literature review. Med. Pharm. Rep..

[53] Sahebi M., Ghodsi S., Berahman P., Amini A., Zeighami S. (2022). Comparison of retention and fracture load of endocrowns made from zirconia and zirconium lithium silicate after aging: An in vitro study. BMC Oral Health.

[54] Schwartz R.S., Robbins J.W. (2004). Post placement and restoration of endodontically treated teeth: A literature review. J. Endod..

[55] Magne P., Goldberg J., Edelhoff D., Güth J.F. (2016). Composite Resin Core Buildups With and Without Post for the Restoration of Endodontically Treated Molars Without Ferrule. Oper. Dent..

[56] Magne P., Lazari P.C., Carvalho M.A., Johnson T., Del Bel Cury A.A. (2017). Ferrule-Effect Dominates Over Use of a Fiber Post When Restoring Endodontically Treated Incisors: An In Vitro Study. Oper. Dent..

[57] Zicari F., Van Meerbeek B., Scotti R., Naert I. (2013). Effect of ferrule and post placement on fracture resistance of endodontically treated teeth after fatigue loading. J. Dent..

[58] Bitter K., Noetzel J., Stamm O., Vaudt J., Meyer-Lueckel H., Neumann K., Kielbassa A.M. (2009). Randomized clinical trial comparing the effects of post placement on failure rate of postendodontic restorations: Preliminary results of a mean period of 32 months. J. Endod..

[59] Dong S., Peng M., Wu G., Yao C., Huang C., Liang S. (2024). Does an incomplete ferrule affect the fracture of endodontically treated teeth? A systematic review of in vitro studies. J. Dent..

[60] Assiri A.Y.K., Saafi J., Al-Moaleem M.M., Mehta V. (2022). Ferrule effect and its importance in restorative dentistry: A literature Review. J. Popul. Ther. Clin. Pharmacol..

[61] Sorensen J.A., Engelman M.J. (1990). Ferrule design and fracture resistance of endodontically treated teeth. J. Prosthet. Dent..

[62] Al-Wahadni A., Gutteridge D.L. (2002). An in vitro investigation into the effects of retained coronal dentine on the strength of a tooth restored with a cemented post and partial core restoration. Int. Endod. J..

[63] Dikbas I., Tanalp J., Ozel E., Koksal T., Ersoy M. (2007). Evaluation of the effect of different ferrule designs on the fracture resistance of endodontically treated maxillary central incisors incorporating fiber posts, composite cores and crown restorations. J. Contemp. Dent. Pract..

[64] Mamoun J.S. (2014). On the ferrule effect and the biomechanical stability of teeth restored with cores, posts, and crowns. Eur. J. Dent..

[65] Dotto L., Girotto L.P.S., Correa Silva Sousa Y.T., Pereira G.K.R., Bacchi A., Sarkis-Onofre R. (2022). Factors influencing the clinical performance of the restoration of endodontically treated teeth: An assessment of systematic reviews of clinical studies. J. Prosthet. Dent..

[66] Al-Sanabani F.A., Al-Makramani B.M., Alaajam W.H., Al-Ak’hali M.S., Alhajj M.N., Nassani M.Z., Assad M., Al-Maweri S.A. (2023). Effect of partial ferrule on fracture resistance of endodontically treated teeth: A meta-analysis of in-vitro studies. J. Prosthodont. Res..

[67] Pinto A.B.A., Andrade GS de Abu Hasna A., Souza JR de Tribst J.P.M., Borges A.L.S. (2024). Can the Remaining Coronal Tooth Structure Influence the Mechanical Behavior of Nonpost Full Crowns?. Eur. J. Dent..

[68] Schmitter M., Hamadi K., Rammelsberg P. (2011). Survival of two post systems—Five-year results of a randomized clinical trial. Quintessence Int..

[69] Torbjörner A., Fransson B. (2004). A literature review on the prosthetic treatment of structurally compromised teeth. Int. J. Prosthodont..

[70] Bergamo E.T.P., Lopes A.C.O., Campos T.M.B., Amorim P.H., Costa F., Benalcázar Jalkh E.B., Carvalho L.F., Zahoui A., Piza M.M., Gutierres E. (2022). Probability of survival and failure mode of endodontically treated incisors without ferrule restored with CAD/CAM fiber-reinforced composite (FRC) post-cores. J. Mech. Behav. Biomed. Mater..

[71] Reddy S.N., Harika K., Manjula S., Chandra P., Vengi L., Koka K.M. (2016). Evaluation of occlusal fracture resistance of three different core materials using the Nayyar core technique. J. Int. Soc. Prev. Community Dent..

[72] van Dijken J.W.V., Pallesen U. (2016). Posterior bulk-filled resin composite restorations: A 5-year randomized controlled clinical study. J. Dent..

[73] Akiya S., Sato K., Kibe K., Tichy A., Hiraishi N., Prasansuttiporn T., Hosaka K., Foxton R.M., Shimada Y., Nakajima M. (2023). Polymerization shrinkage of light-cured conventional and bulk-fill composites-The effect of cavity depth and post-curing. Dent. Mater. J..

[74] Martins L.C., Oliveira L.R.S., Braga S.S.L., Soares C.J., Versluis A., Borges G.A., Verissimo C. (2020). Effect of Composite Resin and Restorative Technique on Polymerization Shrinkage Stress, Cuspal Strain and Fracture Load of Weakened Premolars. J. Adhes. Dent..

[75] Cocco F., Packaeser M.G., Machry R.V., Tribst J.P.M., Kleverlaan C.J., Pereira G.K.R., Valandro L.F. (2024). Conventional-, bulk-fill- or flowable-resin composites as prosthetic core build-up: Influence on the load-bearing capacity under fatigue of bonded leucite-reinforced glass-ceramic. J. Mech. Behav. Biomed. Mater..

[76] Oliveira C.R.d.M., Reis É.G.J., Tanomaru-Filho M., dos Santos Nunes Reis S.N. (2022). Fracture strength of teeth with coronal destruction after core build-up restoration with bulk fill materials. J. Esthet. Restor. Dent..

[77] Zarow M., Dominiak M., Szczeklik K., Hardan L., Bourgi R., Cuevas-Suárez C.E., Zamarripa-Calderón J.E., Kharouf N., Filtchev D. (2021). Effect of Composite Core Materials on Fracture Resistance of Endodontically Treated Teeth: A Systematic Review and Meta-Analysis of In Vitro Studies. Polymers.

[78] Zenthöfer A., Bermejo J.L., Bömicke W., Frese C., Gülmez R., Rammelsberg P., Ohlmann B. (2022). Early failures when using three different adhesively retained core build-up materials—A randomized controlled trial. Clin. Oral Investig..

[79] Selvaraj H., Krithikadatta J., Shrivastava D., Onazi M.A.A., Algarni H.A., Munaga S., Hamza M.O., Al-Fridy T.S., Teja K.V., Janani K. (2023). Systematic review fracture resistance of endodontically treated posterior teeth restored with fiber reinforced composites- a systematic review. BMC Oral Health.

[80] Eskitaşcioğlu G., Belli S., Kalkan M. (2002). Evaluation of two post core systems using two different methods (fracture strength test and a finite elemental stress analysis). J. Endod..

[81] Fousekis E., Lolis A., Marinakis E., Oikonomou E., Foros P., Koletsi D., Eliades G. (2023). Short fiber-reinforced composite resins as post-and-core materials for endodontically treated teeth: A systematic review and meta-analysis of in vitro studies. J. Prosthet. Dent..

[82] Garoushi S., Gargoum A., Vallittu P.K., Lassila L. (2018). Short fiber-reinforced composite restorations: A review of the current literature. J. Investig. Clin. Dent..

[83] Attik N., Colon P., Gauthier R., Chevalier C., Grosgogeat B., Abouelleil H. (2022). Comparison of physical and biological properties of a flowable fiber reinforced and bulk filling composites. Dent. Mater..

[84] Wierichs R.J., Kramer E.J., Wolf T.G., Naumann M., Meyer-Lueckel H. (2019). Longevity of composite build-ups without posts-10-year results of a practice-based study. Clin. Oral Investig..

[85] Zenthöfer A., Rues S., Rammelsberg P., Ohlmann B., Bömicke W. (2023). Influence of geometric dimensions on early failures of adhesively retained composite resin core build-ups. J. Esthet. Restor. Dent..

[86] Cloet E., Debels E., Naert I. (2017). Controlled Clinical Trial on the Outcome of Glass Fiber Composite Cores Versus Wrought Posts and Cast Cores for the Restoration of Endodontically Treated Teeth: A 5-Year Follow-up Study. Int. J. Prosthodont..

[87] Ellner S., Bergendal T., Bergman B. (2003). Four post-and-core combinations as abutments for fixed single crowns: A prospective up to 10-year study. Int. J. Prosthodont..

[88] Ferrari M., Vichi A., García-Godoy F. (2000). Clinical evaluation of fiber-reinforced epoxy resin posts and cast post and cores. Am. J. Dent..

[89] Cheung W. (2005). A review of the management of endodontically treated teeth: Post, core and the final restoration. J. Am. Dent. Assoc..

[90] Bateman G., Ricketts D.N.J., Saunders W.P. (2003). Fibre-based post systems: A review. Br. Dent. J..

[91] Fathi A., Ebadian B., Dezaki S.N., Mardasi N., Mosharraf R., Isler S., Tabatabaei S.S. (2022). An Umbrella Review of Systematic Reviews and Meta-Analyses Evaluating the Success Rate of Prosthetic Restorations on Endodontically Treated Teeth. Int. J. Dent..

[92] Zhou L., Wang Q. (2013). Comparison of Fracture Resistance between Cast Posts and Fiber Posts: A Meta-analysis of Literature. J. Endod..

[93] Opdam N.J.M., van de Sande F.H., Bronkhorst E., Cenci M.S., Bottenberg P., Pallesen U., Gaengler P., Lindberg A., Huysmans M.C.D.N.J.M., van Dijken J.W. (2014). Longevity of posterior composite restorations: A systematic review and meta-analysis. J. Dent. Res..

[94] Da Rosa Rodolpho P.A., Donassollo T.A., Cenci M.S., Loguércio A.D., Moraes R.R., Bronkhorst E.M., Opdam N.J.M., Demarco F.F. (2011). 22-Year clinical evaluation of the performance of two posterior composites with different filler characteristics. Dent. Mater..

[95] Landys Borén D., Jonasson P., Kvist T. (2015). Long-term survival of endodontically treated teeth at a public dental specialist clinic. J. Endod..

[96] Figueiredo F.E.D., Martins-Filho P.R.S., Faria-E-Silva A.L. (2015). Do metal post-retained restorations result in more root fractures than fiber post-retained restorations? A systematic review and meta-analysis. J. Endod..

[97] Martins M.D., Junqueira R.B., de Carvalho R.F., Lacerda M.F.L.S., Faé D.S., Lemos C.A.A. (2021). Is a fiber post better than a metal post for the restoration of endodontically treated teeth? A systematic review and meta-analysis. J. Dent..

[98] Andrade S.A. (2021). Cast metal posts versus glass fibre posts: Which treatment of choice based on cost-minimisation analysis?. Evid. Based Dent..

[99] Gloria A., Maietta S., Richetta M., Ausiello P., Martorelli M. (2019). Metal Posts and the Effect of Material–Shape Combination on the Mechanical Behavior of Endodontically Treated Anterior Teeth. Metals.

[100] Kharouf N., Sauro S., Jmal H., Eid A., Karrout M., Bahlouli N., Haikel Y., Mancino D. (2021). Does Multi-Fiber-Reinforced Composite-Post Influence the Filling Ability and the Bond Strength in Root Canal?. Bioengineering.

[101] Santos T.d.S.A., Abu Hasna A., Abreu R.T., Tribst J.P.M., de Andrade G.S., Borges A.L.S., Torres C.R.G., Carvalho C.A.T. (2022). Fracture resistance and stress distribution of weakened teeth reinforced with a bundled glass fiber–reinforced resin post. Clin. Oral Investig..

[102] Haralur S.B., Al Ahmari M.A., AlQarni S.A., Althobati M.K. (2018). The Effect of Intraradicular Multiple Fiber and Cast Posts on the Fracture Resistance of Endodontically Treated Teeth with Wide Root Canals. BioMed Res. Int..

[103] Richert R., Robinson P., Viguie G., Farges J.C., Ducret M. (2018). Multi-Fiber-Reinforced Composites for the Coronoradicular Reconstruction of Premolar Teeth: A Finite Element Analysis. BioMed Res. Int..

[104] Alkhalidi E. (2020). Fracture Resistance of New Fiber Post System (Rebilda GT). Indian J. Forensic Med. Toxicol..

[105] Yanık D., Turker N. (2022). Stress distribution of a novel bundle fiber post with curved roots and oval canals. J. Esthet. Restor. Dent..

[106] Sturm R., Prates Soares A., Sterzenbach G., Bitter K. (2021). Interface analysis after fatigue loading of adhesively luted bundled fiber posts to human root canal dentin. J. Mech. Behav. Biomed. Mater..

[107] Bitter K., Falcon L., Soares A.P., Sturm R., von Stein-Lausnitze M., Sterzenbach G. (2019). Effect of Application Mode on Bond Strength of Adhesively Luted Glass-fiber Bundles Inside the Root Canal. J. Adhes. Dent..

[108] Kul E., Yanıkoğlu N., Yeşildal Yeter K., Bayındır F., Sakarya R.E. (2020). A comparison of the fracture resistance of premolars without a ferrule with different post systems. J. Prosthet. Dent..

[109] Ranjkesh B., Haddadi Y., Krogsgaard C.A., Schurmann A., Bahrami G. (2022). Fracture resistance of endodontically treated maxillary incisors restored with single or bundled glass fiber-reinforced composite resin posts. J. Clin. Exp. Dent..

[110] Setzer F.C., Kratchman S.I. (2022). Present status and future directions: Surgical endodontics. Int. Endod. J..

[111] Mahmoudi M., Saidi A.R., Amini P., Hashemipour M.A. (2017). Influence of inhomogeneous dental posts on stress distribution in tooth root and interfaces: Three-dimensional finite element analysis. J. Prosthet. Dent..

[112] Gloria A., Maietta S., Martorelli M., Lanzotti A., Watts D.C., Ausiello P. (2018). FE analysis of conceptual hybrid composite endodontic post designs in anterior teeth. Dent. Mater..

[113] Sorrentino R., Di Mauro M., Ferrari M., Leone R., Zarone F. (2016). Complications of endodontically treated teeth restored with fiber posts and single crowns or fixed dental prostheses-a systematic review: Clinical Oral Investigations. Clin. Oral Investig..

[114] Onay E.O., Korkmaz Y., Kiremitci A. (2010). Effect of adhesive system type and root region on the push-out bond strength of glass-fibre posts to radicular dentine. Int. Endod. J..

[115] Marques de Melo R., Galhano G., Barbosa S.H., Valandro L.F., Pavanelli C.A., Bottino M.A. (2008). Effect of adhesive system type and tooth region on the bond strength to dentin. J. Adhes. Dent..

[116] Ohlmann B., Fickenscher F., Dreyhaupt J., Rammelsberg P., Gabbert O., Schmitter M. (2008). The effect of two luting agents, pretreatment of the post, and pretreatment of the canal dentin on the retention of fiber-reinforced composite posts. J. Dent..

[117] Wang V.J.J., Chen Y.M., Yip K.H.K., Smales R.J., Meng Q.F., Chen L. (2008). Effect of two fiber post types and two luting cement systems on regional post retention using the push-out test. Dent. Mater..

[118] Mobilio N., Borelli B., Sorrentino R., Catapano S. (2013). Effect of fiber post length and bone level on the fracture resistance of endodontically treated teeth. Dent. Mater. J..

[119] Matsumoto M., Miura J., Takeshige F., Yatani H. (2013). Mechanical and morphological evaluation of the bond-dentin interface in direct resin core build-up method. Dent. Mater..

[120] de Morais D.C., Butler S., Santos M.J.M.C. (2023). Current Insights on Fiber Posts: A Narrative Review of Laboratory and Clinical Studies. Dent. J..

[121] Maravić T., Mazzitelli C., Mancuso E., Del Bianco F., Josić U., Cadenaro M., Breschi L., Mazzoni A. (2023). Resin composite cements: Current status and a novel classification proposal. J. Esthet. Restor. Dent..

[122] Farina A.P., Cecchin D., da Fonseca Roberti Garcia L., Naves L.Z., de Carvalho Panzeri Pires-de-Souza F. (2011). Bond strength of fibre glass and carbon fibre posts to the root canal walls using different resin cements. Aust. Endod. J..

[123] Sterzenbach G., Karajouli G., Naumann M., Peroz I., Bitter K. (2012). Fiber post placement with core build-up materials or resin cements-an evaluation of different adhesive approaches. Acta Odontol. Scand..

[124] Angnanon W., Thammajaruk P., Guazzato M. (2023). Effective luting agents for glass-fiber posts: A network meta-analysis. Dent. Mater..

[125] Foxton R.M., Nakajima M., Tagami J., Miura H. (2003). Bonding of photo and dual-cure adhesives to root canal dentin. Oper. Dent..

[126] Abdel-Gawad S., Dursun E., Ceinos R., Le Goff S., Fasham T., Attal J.P., Francois P. (2024). Touch-cure activation by marketed universal resin luting cements of their associated primer to dentin. J. Oral Sci..

[127] Jurema A.L.B., Correia A.M.D.O., Spinola M.D.S., Bresciani E., Caneppele T.M.F. (2022). Influence of different intraradicular chemical pretreatments on the bond strength of adhesive interface between dentine and fiber post cements: A systematic review and network meta-analysis. Eur. J. Oral Sci..

[128] Knight B., Love R.M., George R. (2018). Evaluation of the influence of time and concentration of sodium hypochlorite on the bond strength of glass fibre post. Aust. Endod. J..

[129] Dilts W.E., Miller R.C., Miranda F.J., Duncanson M.G. (1986). Effect of zinc oxide-eugenol on shear bond strengths of selected core/cement combinations. J. Prosthet. Dent..

[130] Klosa K., Shahid W., Aleknonytė-Resch M., Kern M. (2020). Cleaning and Conditioning of Contaminated Core Build-Up Material before Adhesive Bonding. Materials.

[131] Plotino G., Abella Sans F., Duggal M.S., Grande N.M., Krastl G., Nagendrababu V., Gambarini G. (2022). Present status and future directions: Surgical extrusion, intentional replantation and tooth autotransplantation. Int. Endod. J..

[132] Reichardt E., Krug R., Bornstein M.M., Tomasch J., Verna C., Krastl G. (2021). Orthodontic Forced Eruption of Permanent Anterior Teeth with Subgingival Fractures: A Systematic Review. Int. J. Environ. Res. Public Health.

[133] Pilalas I., Tsalikis L., Tatakis D.N. (2016). Pre-restorative crown lengthening surgery outcomes: A systematic review. J. Clin. Periodontol..

[134] Samartzi T.K., Papalexopoulos D., Ntovas P., Rahiotis C., Blatz M.B. (2022). Deep Margin Elevation: A Literature Review. Dent. J..

[135] Dietschi D., Spreafico R. (1998). Current clinical concepts for adhesive cementation of tooth-colored posterior restorations. Pract. Periodontics Aesthet. Dent..

[136] Juloski J., Köken S., Ferrari M. (2018). Cervical margin relocation in indirect adhesive restorations: A literature review. J. Prosthodont. Res..

[137] Falahchai M., Musapoor N., Mokhtari S., Babaee Hemmati Y., Neshandar Asli H. (2023). Fracture resistance and failure mode of endodontically treated premolars reconstructed by different preparation approaches: Cervical margin relocation and crown lengthening with complete and partial ferrule with three different post and core systems. J. Prosthodont..

[138] Bresser R.A., Gerdolle D., van den Heijkant I.A., Sluiter-Pouwels L.M.A., Cune M.S., Gresnigt M.M.M. (2019). Up to 12 years clinical evaluation of 197 partial indirect restorations with deep margin elevation in the posterior region. J. Dent..

[139] Vertolli T.J., Martinsen B.D., Hanson C.M., Howard R.S., Kooistra S., Ye L. (2020). Effect of Deep Margin Elevation on CAD/CAM-Fabricated Ceramic Inlays. Oper. Dent..

[140] Bresser R.A., van de Geer L., Gerdolle D., Schepke U., Cune M.S., Gresnigt M.M.M. (2020). Influence of Deep Margin Elevation and preparation design on the fracture strength of indirectly restored molars. J. Mech. Behav. Biomed. Mater..

[141] Eggmann F., Ayub J.M., Conejo J., Blatz M.B. (2023). Deep margin elevation-Present status and future directions. J. Esthet. Restor. Dent..

[142] Sarfati A., Tirlet G. (2018). Deep margin elevation versus crown lengthening: Biologic width revisited. Int. J. Esthet. Dent..

[143] Ferrari M., Koken S., Grandini S., Ferrari Cagidiaco E., Joda T., Discepoli N. (2018). Influence of cervical margin relocation (CMR) on periodontal health: 12-month results of a controlled trial. J. Dent..

[144] Farouk A.T., Hassanein O.E.S., Fahmy O.I., Elkady A.M., ElNahass H. (2023). Biological evaluation of indirect restorations in endodontically treated posterior teeth with deeply located proximal margins following deep margin elevation versus surgical crown lengthening: A randomized controlled trial. Clin. Oral. Investig..

[145] Ilgenstein I., Zitzmann N.U., Bühler J., Wegehaupt F.J., Attin T., Weiger R., Krastl G. (2015). Influence of proximal box elevation on the marginal quality and fracture behavior of root-filled molars restored with CAD/CAM ceramic or composite onlays. Clin. Oral Investig..

[146] Grubbs T.D., Vargas M., Kolker J., Teixeira E.C. (2020). Efficacy of Direct Restorative Materials in Proximal Box Elevation on the Margin Quality and Fracture Resistance of Molars Restored With CAD/CAM Onlays. Oper. Dent..

[147] Baldi A., Scattina A., Ferrero G., Comba A., Alovisi M., Pasqualini D., Peroni L., Muggeo M., Germanetti F., Scotti N. (2022). Highly-filled flowable composite in deep margin elevation: FEA study obtained from a microCT real model. Dent. Mater..

